# RNAmod: an integrated system for the annotation of mRNA modifications

**DOI:** 10.1093/nar/gkz479

**Published:** 2019-05-31

**Authors:** Qi Liu, Richard I Gregory

**Affiliations:** 1 Stem Cell Program, Division of Hematology/Oncology, Boston Children's Hospital, Boston, MA 02115, USA; 2 Department of Biological Chemistry and Molecular Pharmacology, Harvard Medical School, Boston, MA 02115, USA; 3 Department of Pediatrics, Harvard Medical School, Boston, MA 02115, USA; 4 Harvard Initiative for RNA Medicine, Boston, MA 02115, USA; 5 Harvard Stem Cell Institute, Cambridge, MA 02138, USA

## Abstract

Dynamic and reversible RNA modifications such as *N*^6^-methyladenosine (m^6^A) can play important roles in regulating messenger RNA (mRNA) splicing, export, stability and translation. Defective mRNA modification through altered expression of the methyltransferase and/or demethylases results in developmental defects and cancer progression. Identifying modified mRNAs, annotating the distribution of modification sites across the mRNA, as well as characterizing and comparing other modification features are essential for studying the function and elucidating the mechanism of mRNA modifications. Several methods including methylated RNA immunoprecipitation and sequencing (MeRIP-seq) are available for the detection of mRNA modifications. However, a convenient and comprehensive tool to annotate diverse kinds of mRNA modifications in different species is lacking. Here, we developed RNAmod (https://bioinformatics.sc.cn/RNAmod), an interactive, one-stop, web-based platform for the automated analysis, annotation, and visualization of mRNA modifications in 21 species. RNAmod provides intuitive interfaces to show outputs including the distribution of RNA modifications, modification coverage for different gene features, functional annotation of modified mRNAs, and comparisons between different groups or specific gene sets. Furthermore, sites of known RNA modification, as well as binding site data for hundreds of RNA-binding proteins (RBPs) are integrated in RNAmod to help users compare their modification data with known modifications and to explore the relationship with the binding sites of known RBPs. RNAmod is freely available and meets the emerging need for a convenient and comprehensive analysis tool for the fast-developing RNA modification field.

## INTRODUCTION

There are over 160 known RNA modifications and together this repertoire of different modifications has been collectively termed the ‘epitranscriptome’ (1). Several of these modifications have been identified in mRNA, indicating possible roles in regulating gene expression (2). For example *N*^6^-methyladenosine (m^6^A), the most abundant modification on mRNA, has been linked to the control of mRNA splicing, export, stability, and translation (3). m^6^A modification can be dynamically and reversibly regulated by the action of the RNA methyltransferase METTL3 and demethylases ALKBH5 and FTO (4). Accordingly, mis-regulation of mRNA modifications results in multi-tissue developmental defects and cancer progression (5–9). Modifications on mRNAs are not randomly distributed, rather they occur on specific sequence motifs at certain locations on mRNA. For example, m^6^A is mainly located near the translation start codon and translation stop codon (10); while N^1^-methyladenosine (m^1^A) is highly enriched at the 5’ untranslated region (UTR) of mRNA (11,12). Additional mRNA modifications have recently been identified, including cytidine *N*^4^-acetylation (ac^4^C) (13) and internal *N*^7^-methylguanosine (m^7^G) (14).

The localization of mRNA modification correlates with its molecular functions, therefore, determining the distribution of modification across the mRNA and annotating the modified gene targets are essential for studying the function of modifications. Next-generation sequencing (NGS) based technologies has offered an unprecedented opportunity to discover and profile RNA modifications under various conditions, such as MeRIP-seq (methylated RNA immunoprecipitation and sequencing (15), miCLIP-seq (m^6^A individual-nucleotide-resolution cross-linking and immunoprecipitation and sequencing) (16) and detection of m^7^G by TRAC-seq (tRNA reduction and cleavage-sequencing) (17) and m^7^G-seq (14). Meanwhile, several prediction tools based on machine learning were developed to systematically predict RNA modifications, including WHISTLE (18), SRAMP (19), M6AMRFS (20), RAM-ESVM (21), DeepM6ASeq (22). All these methods have greatly facilitated the identification of mRNA modifications. However, the detailed annotation of these dynamic modifications is still a bottleneck for better understanding of their function. There are some tools available for the annotation of RNA modifications, such as RNAModR (23) and Guitar (24) that generate meta-gene plots of modification sites/peaks, and RCAS (25) for meta-gene plots and RNA based annotations, and some databases, such as RMBase (26) also provide the function to perform meta-gene plots. However, to the best of our knowledge, all available tools pay more attention to conducting just meta-gene analysis and there is no convenient web server available for the automated analysis and annotation of mRNA modifications. A comprehensive web-based tool to annotate diverse kinds of mRNA modifications and their potential functions for different species is still lacking.

Here, we developed RNAmod, a very convenient web-based platform, for the analysis and functional annotation of mRNA modifications in up to 21 species, including human, mouse, rat, zebrafish, fly, yeast and Arabidopsis, etc. RNAmod uses the commonly used BED format of chromosomal location of RNA modifications as input, which can be generated by almost all site/peak calling tools, such as MACS (27), exomePeak (28) and BayesPeak (29). RNAmod first maps individual modification sites onto RNAs and then performs various analysis and annotations, including peak/site distribution among different RNA features, distribution among different gene biotypes, peak/site coverage for different gene features, coverage around transcription start/end sites and translation start/end sites, coverage around splicing sites, mRNA metagene analysis, and gene characteristics and functional analyses of modified mRNAs, thereby providing a comprehensive annotation and visualization of the distribution of mRNA modifications. Besides, RNAmod allows the user to perform comparative analysis between groups or between specific gene sets and other background genes. Moreover, JBrowse is integrated in RNAmod and contains known modification sites and binding sites of RBP, which will help users to compare their modification sites with known modifications and RBP binding sites. It is noted that RNAmod can also be used for the annotation and visualization of CLIP-seq data to study the binding features of RBPs. In the download page, RNAmod provides the annotation for known modification sites and binding sites of nearly 500 RBPs from different species. The web server pages of RNAmod utilize several intuitive JavaScript libraries to show the results in very interactive ways. Meanwhile, users can download all the outputs using a one-click link. Overall, RNAmod is a one-stop online interactive platform for the annotation, analysis, and visualization of mRNA modifications. The website is freely available and open access with no login required (https://bioinformatics.sc.cn/RNAmod or http://61.147.117.195/RNAmod or https://rnabioinfor.tch.harvard.edu/RNAmod). We believe that it will meet the emerging need for a convenient and comprehensive analysis tool for the fast-developing RNA biology research field.

## ANALYSIS WORKFLOW

### Data sources

The gene annotation files in GTF format and mRNA transcript sequences in FASTA format were downloaded from GENCODE (https://www.gencodegenes.org) for human (hg38 and hg19) and mouse (mm10), while for the other 19 species, the annotation files were retrieved from FTP site of Ensembl genome database (30) in which, compared with other databases, the annotations contain not only mRNA but also other types of RNAs. To accelerate data processing and gene features extraction in the RNAmod web server, all GTF gene annotations were stored in TxDB objects in the SQLite database by using GenomicFeatures (31) and RSQLite packages in R (https://www.r-project.org). The reference genomes used for the sequences extraction were stored as BSgenome packages. For the species without an available BSgenome package in Bioconductor (https://www.bioconductor.org), we constructed them using in-house developed programs based on genome sequence download from Ensembl. We split the mRNA sequences into three different parts (5′ UTR, CDS and 3′ UTR) and calculated their gene characteristics separately. The characteristics include sequence length, GC content, and minimum free energy, which are used in the comparative characteristics analysis of modified genes. RNAFold (32) was used to calculate the minimum free energy. For the functional annotation of modified genes, the Gene Ontology (GO) terms and KEGG pathways are provided by clusterProfiler package (33), Reactome pathways are packaged with ReactomePA packages (34), while Disease Ontology, Network of Cancer Gene and DisGeNET disease genes are available in DOSE packages (35). Other gene sets used in functional enrichments include chemical and genetic perturbation genes, microRNA target motif genes, cancer gene neighborhoods, cancer module genes, oncogenic signature genes, and immunologic signature genes, which are all downloaded from The Molecular Signatures Database (MSigDB) (36). The known RNA modification sites data were obtained from RMBase 2.0 (26) and MeT-DB V2.0 (37) while RBP binding sites data were obtained from POSTAR (38) database. The known RNA modification sites in RMBase contain different kinds of modification sites identified from a large number of high-throughput sequencing studies. We separated them into individual files by study and samples. MeT-DB V2.0 is an important database for m^6^A modification and contains 185 samples from 26 independent studies for seven species. RBP binding site information in POSTAR database are already organized by samples. The modification sites information and RBP binding site data for each sample were transformed into BED format, which were then stored in JBrowse using flatfile-to-json.pl script and annotated using RNAmod.

### RNA modification data processing in RMBase 2.0 and Met-DB 2.0

In RMBase 2.0 (26), raw sequencing data of m^6^A-seq or MeRIP-seq is obtained from the Gene Expression Omnibus (GEO) (39) and the Sequence Read Archive (SRA) (40) were first cleaned using cutadapt (https://cutadapt.readthedocs.io/en/stable) and FASTX-toolkit (http://hannonlab.cshl.edu/fastx_toolkit). The clean data were then mapped to the reference genomes using hisat2 (41) with default parameters and the converted BAM mapping results were used to call modification peaks using exomePeak (28) with the parameters ‘fold change (FC) >2, *P*-value <0.01 and false discovery rate (FDR) <0.05’. Based on the identified peaks, the exact m^6^A positions were predicted by searching consensus RRACH motifs (R denotes A or G while H represents A, C or U) (16). By using a similar strategy, the m^1^A modification sites were detected based on the peaks from m^1^A-seq using GAAGAAG motif (42). The peaks/sites of other less abundant modifications, such as pseudouridine and m^5^C, were manually collected from the respective studies. In Met-DB 2.0, the raw sequencing data of MeRIP-seq IP/Input collected from SRA (40) were cleaned using Trim Galore (v0.4.2) (https://www.bioinformatics.babraham.ac.uk/projects/trim_galore) and mapped to the reference genomes using Tophat2 (v2.1.0) (43) with default parameters. Based on the BAM mapping files of IP and input samples, m^6^A peaks were then called using exomePeak (28) with default parameters.

### Overview of RNAmod

**Figure 1.**
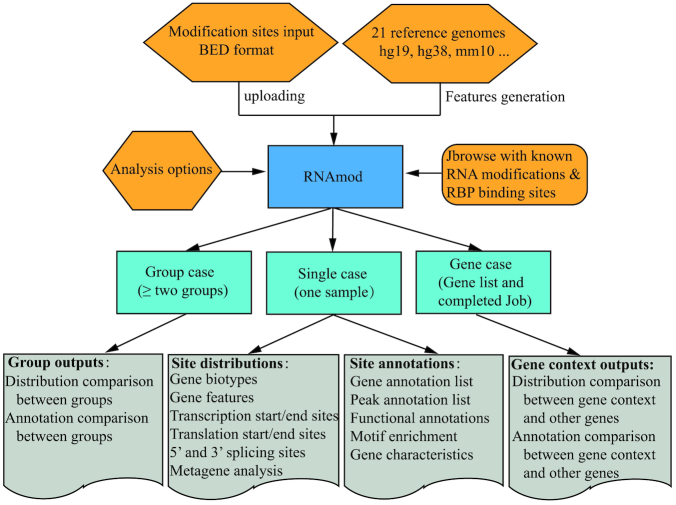
Overall workflow of RNAmod. RNAmod provides three functional modules, including single case, group case, and gene case. The single case module allows users to annotate RNA modifications for a single sample. The group case module allows users to annotate and compare the modification distribution between two samples or even more groups. The gene case analysis module can be used to analyze the modification distribution in the context of specific genes.

The overall workflow of RNAmod is shown in Figure 1. RNAmod first extracts gene features from the reference genome annotation and then maps the submitted modification sites onto different RNA features, it then performs various coverage calculations, metagene analyses, and annotations focusing on mRNAs. The annotations include: (i) site distribution among different gene features and gene biotypes, (ii) coverage analysis among RNA features, (iii) site distribution around transcription start/end site, (iv) site distribution around translation start/end sites, (v) site distribution around splicing junction sites, (vi) comparison of gene characteristics between modified genes and other genes, (vii) modified site heatmap around translation start/end sites and transcription start/end sites, (viii) mRNA metagene analysis, (ix) motif enrichment analysis and (x) functional enrichments for modified genes. To facilitate users who have different analysis requirements, the web server of RNAmod provides three functional modules, including single case, group case and gene case. The single case module allows users to annotate RNA modifications for a single sample. The group case modules allow users to annotate and compare the distribution of modifications between two samples or even more groups. Considering the situation in which users need to know the modification site distribution and modified gene features for a specific gene or set of genes, such as translation down-regulated genes, cell-cycle genes, or neural development genes, the gene analysis module can be used to achieve this goal by allowing users to input a specific gene group. Meanwhile, we integrated JBrowse tool (44) to help users to check and compare the modification in the context of known RNA modification sites and RNA-binding sites of RBPs.

### Data input of RNAmod

**Figure 2.**
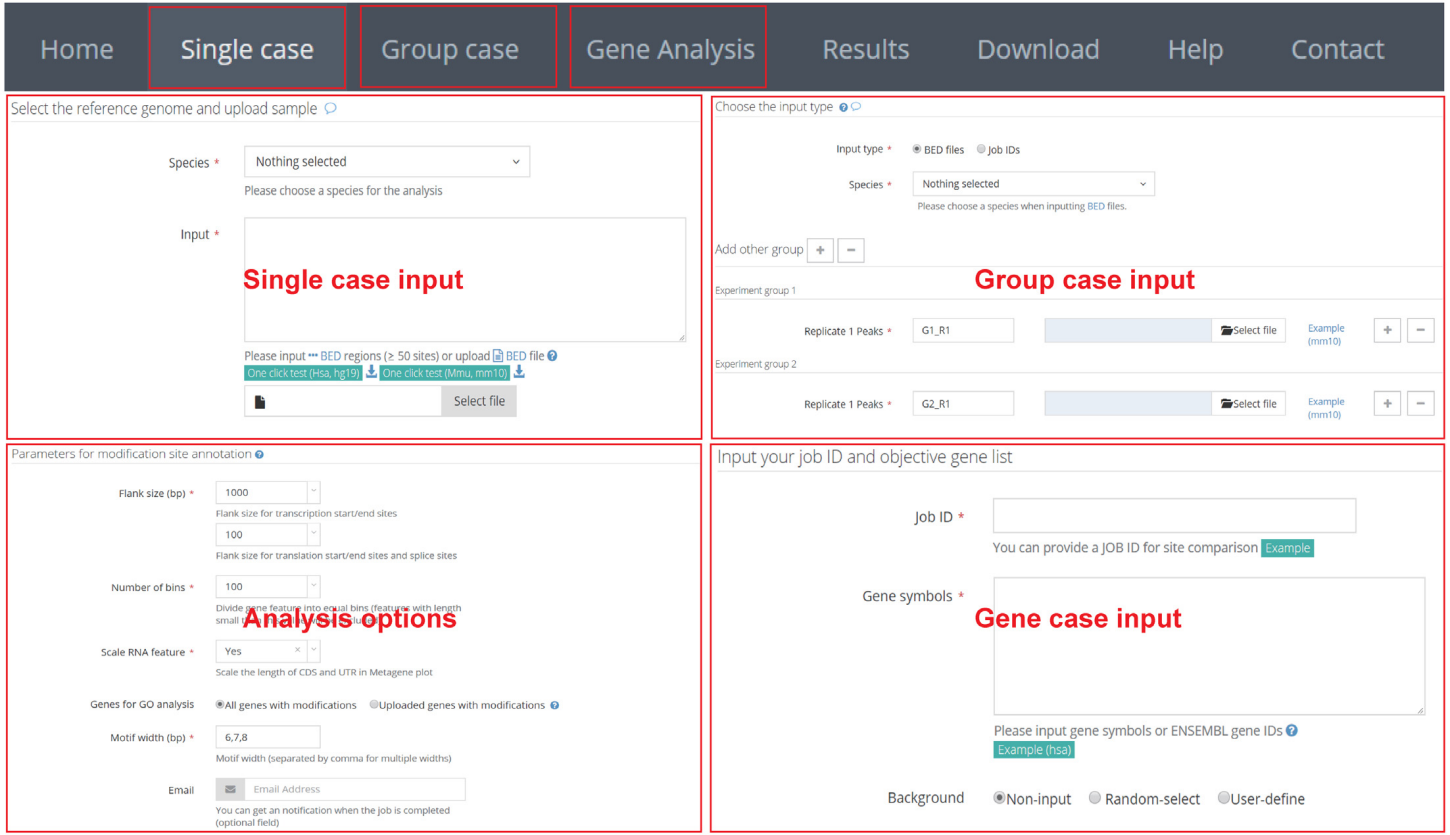
Inputs of RNAmod. In the single case input, RNAmod utilizes the commonly used BED format of the chromosomal location of RNA modifications as input. In the group case, two types of inputs are supported: BED format files and single case job IDs. In the gene case input, users are required to input the single case or group case job IDs and the object gene list.

In the single case, RNAmod utilizes the commonly used BED format of chromosomal location of RNA modifications as input, which can be generated by almost all modification sites/peak calling tools based on MeRIP-seq, such as MACS (27), exomePeak (28), BayesPeak (29) and MetPeak (45). BED format can also be easily converted from other text outputs that are generated from the data analyses of other protocols, such as miCLIP-m6A (16) and Pseudo-Seq (46). In the links web page, RNAmod also provides an R script for users to call the modification peaks/sites from MeRIP-seq data. To speed up the uploading, users are recommended to submit a compressed BED file in .gz or .zip format. After submission of data, the data analysis queue system provides users with a job ID, a string consisting of 16 random characters, which can be used to retrieve the results once the job is finished. In the group case module, two types of inputs are supported: BED format files and single case job IDs. When a single case analysis job ID is given, the web server will retrieve the completed annotation automatically. In the gene case, users are required to input the single case or group case job IDs, and the gene list (Figure 2).

RNAmod provides users several parameters to flexibly re-define the mRNA features. The first is flank length interval (upstream and downstream), which can be used to define the transcription site/end site genomic regions, translation start/end site, and splicing site transcript regions. The second is the number of bins in which to divide the gene features for coverage plots and mRNA metagene analyses. The third is the parameter controlling the shape of metagene plot curve. The fourth is the length of the motif in the enriched motif identification. Meanwhile, RNAmod allows users to submit a group of genes to define the subset of modified genes used to conduct functional annotations. In the group case, an additional parameter is provided to control whether the intersection or union set in each group is used in the annotation. Notably, all input webpages are organized with examples to help users with correct inputs.

### Annotation and analysis workflow

RNAmod first extracts different gene features from the reference genome annotations, including (i) genomic regions of 5’UTR, CDS, 3’UTR, promoter regions, downstream regions, transcription start sites; (ii) transcript regions of translation start/end site and splicing sites; (iii) gene characteristics of GC content, length and minimum free energy. Next, based on extracted gene features, RNAmod performs various calculations based on the genomic locations of uploaded modification sites, including the intersection with different gene biotypes, intersection with gene features, modification distribution around specific site boundaries, and motif enrichment for the peaks/locations. For metagene analyses for each mRNA feature or the whole mRNA, after excluding the features with length shorter than the bins number, each gene feature is divided into equal size bins (100 bins by default) and the average number of sites is counted for each bin. For the coverage distribution near specific site boundaries, including transcription start/end sites, translation start/end site, and splicing site, RNAmod calculates the average coverage of peaks/sites for each nucleotide both upstream and downstream of the location. For motif analysis, sequences around modification sites and random genomic regions are extracted from constructed BSgenome package using getSeq function in Biostrings package. Then the extracted sequences are used as input for Homer software (47) (http://homer.ucsd.edu/homer) to identify enriched motifs (with motifs lengths of 6, 7 and 8 bp set as default). Finally, based on the genes with modifications or the gene subset intersected from a user submitted list, RNAmod performs various functional annotations, including GO and KEGG pathway for all supported species, and Reactome pathway, Disease Ontology, Network of Cancer Gene, DisGeNET disease genes and MSigDB functional gene sets for human, mouse, rat, zebrafish, fly and C.elegans.

### The output of RNAmod

**Figure 3.**
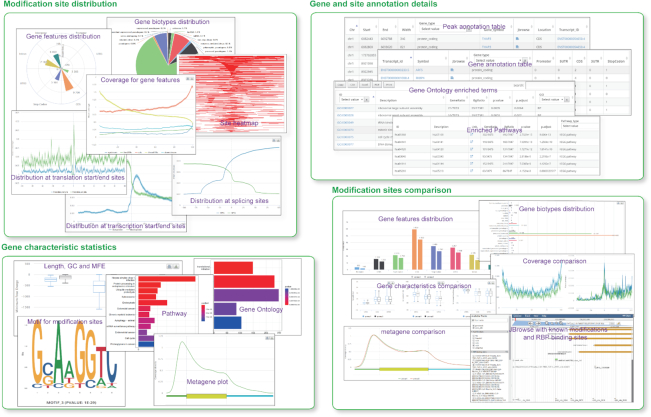
Screenshots of the RNAmod outputs. The typical outputs contain four types of information: the distribution of modification sites, statistics of gene characteristics, gene and site annotation details, and comparison of modification sites.

The outputs of RNAmod are presented in intuitive web interfaces, which typically contain the following types of information: (i) The overall statistics of modifications across different RNA features, with the default priority of CDS, 3’ UTR, 5’ UTR, intron, promoter, and downstream if overlap annotations are found. (ii) The overall statistics of modifications across different RNA biotypes, such as mRNA, miRNA, tRNA, mt-tRNA, and lincRNA. (iii) Plot of modification site coverage at mRNA transcription start/end sites (with 1000 bp flank size as default). (iv) Plot of modification site coverage around translation start/end sites (with 100 bp flank size as default). (v) Plot of modification site coverage at the mRNA 5’ and 3’ exon/intron splicing junction sites (with 100 bp flank size as default). (vi) The coverage of modification sites among different gene features, including promoter, 5’ UTR, CDS, 3’UTR and downstream regions. (vii) The metagene plot of modification sites across whole mRNA. (viii) The top five enriched motifs for modification sites. (ix) The density heatmaps of modifications across transcription start/end site regions. (x) The density heatmaps of modifications across translation start/end site regions. (xi) The GO functional annotation barplots and detailed list of enriched GO terms. (xiii) The pathway functional annotation barplots and detailed list of enriched pathways. (xiii) Detailed lists of modified genes and modification sites. (xiv) JBrowse integration for the visualization of modification sites in comparison to known RNA modifications and RBP binding sites. In the coverage plots, 95% confidence interval (mean ± standard error of the mean × 1.96) is also shown. In the group-case study, besides the basic statistics and site annotation list for each group, the outputs also contain statistics for modification distribution comparison and the overlapping modification sites between groups. In the gene-case study, instead of the comparison between groups, the comparison between input genes and other background genes will be performed, with the output results shown in a similar format to that in the group case. All of the results are shown in interactive tables and figures on the web page, and these are all available for downloading as different formats (Figure 3). It is also noted that RNAmod provides users different ways to show the distribution charts and provides a link to download all the results.

### Implementation

The web server is hosted within PHP/Apache environment under a Linux system and is equipped with two Octa-cores AMD processors (2.6 GHz each) and 64GB of RAM. The back-end pipeline is implemented in the Python/Perl language, and the plots are drawn by R (http://www.r-project.org). Several JavaScript libraries, such as JQuery, Bootstrap.js, DataTable.js, and Highchart.js libraries, were used for creating dynamic and interactive data visualization web browser interfaces, which provide users with a highly intuitive interface for manipulating the tool and viewing the analysis results.

### Comparison with other web servers

There are several web servers that are based on machine-learning technologies for identifying diverse mRNA modifications, especially m^6^A (Supplementary Table S1). For example, SRAMP (19) uses a machine-learning framework to predict mammalian m^6^A sites by extracting and integrating the sequence and structural features around m^6^A sites. HAMR (48) is a method to detect and differentiate different classes of modifications in RNA-seq data based on the principle that RNA modification site can affect RT-activity that leads to sequence mismatches or truncations. RNAm5Cfinder (49) is a web-server that uses Random Forest and RNA sequence features to predict RNA m^5^C modification sites in mouse and human. However, there is still a lack of a convenient and comprehensive web server for the downstream annotation of mRNA modifications. Although some tools or databases provide modification annotation functions (Supplementary Table S1), their functionality is still limited. For instance, RMBase (26) is a comprehensive database for RNA modifications and their associations with post-transcriptional regulation and it provides a convenient web tool—modTools (modMetagene and modAnnotation) (26) to annotate modification sites. modMetagene can be used to draw the metagene plot while modAnnotation displays the table of modification site with gene annotations. Both available annotation tools pay more attention to conducting meta-gene analysis and basic gene annotations.

## CASE STUDIES

RNAmod was used to annotate five mRNA modifications, including N6-methyladenosine (m^6^A), *N*^1^-methyladenosine (m^1^A), 5-methylcytosine (m^5^C), pseudouridine (φ) and *N*^4^-acetylcytidine (ac^4^C) modifications (Supplementary Figure S1). The results showed that the m^6^A sites are mainly located within the CDS and UTRs, with a density peak near the translation end sites (stop codons). Other modifications show similar distribution of sites with enrichment near the translation start sites (start codons), especially ac^4^C modification, which is in accordance with their reported roles in regulating translation. In contrast, a large proportion of pseudouridine modifications are located in 3’UTR regions, which may indicate specific functions in mRNA regulation.

RNAmod can also be used to conveniently annotate RBP binding sites and analyze their binding patterns. Insulin-like growth factor 2 mRNA-binding proteins (IGF2BPs) have been identified as m^6^A readers that can guard thousands of mRNA transcripts from decay (50). RNAmod annotation of IGF2BP3 (a member of IGF2BP gene family) binding sites showed that it mainly binds to 3’UTR regions of protein coding genes (Supplementary Figure S2A, S2B and S2G) with a GGATG motif (Supplementary Figure S2H). The coverage plot, metagene plot, and heatmaps all show that the binding sites showed obvious peak density near the stop codon (Supplementary Figure S2C, S2E and S2I). Differential binding was observed at exons and intron at splice sites (Supplementary Figure S2D). The gene characteristics comparison between IGF2BP3 binding genes and background genes indicates that the 3’UTR of binding genes have longer length and less minimum free energy. The visualization of a specific mRNA example using Jbrowse further showed that the IGF2BP3 binding sites are in close proximity to the known m^6^A modification site (Supplementary Figure S2J).

### Perspective

High-throughput sequencing greatly facilitates RNA epitranscriptomics studies and offers an effective method to comprehensively investigate mRNA modification in genome scale. Meanwhile, many software or web servers are developed to predict mRNA modification using machine-learning technologies. However, the convenient and integrated annotation of these mRNA modifications from large amounts of data still remains a challenge. Therefore, we developed an automated and easy-to-use web service, RNAmod, for research communities to analyze and annotate mRNA modifications. Currently, RNAmod supports 21 model reference genomes across vertebrates, insects, nematodes and plants. More species will be supported in the further. Besides, the data in RNAmod will be updated regularly to keep up with the source databases.

Meanwhile, RNAmod can also be used for the annotation and visualization of CLIP-Seq data to study the binding features of RNA binding proteins (RBPs). We used RNAmod to annotate the known RNA modification sites and nearly 500 RBP binding sites for human, mouse, fly, yeast, zebrafish, Arabidopsis, etc., whose results are availed in the download page of RNAmod. In addition, RNAmod allows users to perform comparative analysis between groups or among specific gene contexts. Overall, RNAmod is a one stop online interactive platform for the annotation, analysis, and visualization of mRNA modifications. It will meet the emerging need for a convenient, and comprehensive analysis tool for the research field.

## Supplementary Material

gkz479_Supplemental_Files
