# Supplementary material for: RNAmod: an integrated system for the annotation of mRNA modifications
Source: Nucleic Acids Res. 2019 May 31;47(W1):W548–55. doi: 10.1093/nar/gkz479 (PMC6602476; doi:10.1093/nar/gkz479)
Supplement: gkz479_Supplemental_Files [file gkz479_supplemental_files.zip › Supplementary Data.pdf]

Figure S1. RNAmod annotation for five mRNA modifications, including N6-methyladenosine (m^6A), N1-methyladenosine (m^1A), 5-methylcytosine (m^5C), pseudouridine (φ) and N4-acetylcytidine (ac^4C) modifications. (**A**) Distribution of individual modifications across gene features. (**B**) Metagene analysis of the distribution of individual modifications.

Figure S2. RNAmod annotation for the m^6A reader protein IGF2BP3. (**A**) Distribution of IGF2BP3 binding across gene features. (**B**) Coverage plot of IGF2BP3-binding for different gene features. (**C**) Coverage plot of IGF2BP3-binding near the translation start site (TSS) and translation end sites (TES). (**D**) Coverage plot of IGF2BP3-binding near the 5' splice sites (5PSS) and 3' splice sites (3PSS). (**E**) Meta-gene plot of IGF2BP3-binding. (**F**) Comparison of gene characteristics between IGF2BP3 bound mRNAs and background genes. (**G**) Distribution of IGF2BP3 binding sites among different RNA biotypes. (**H**) Enriched motif in IGF2BP3 bound mRNAs (**I**) Heatmap showing IGF2BP3 binding near the translation start site (TSS) and translation end sites (TES). (**J**) JBrowse used to compare the binding of IGFBP3 and sites of m^6A modification at a specific gene.

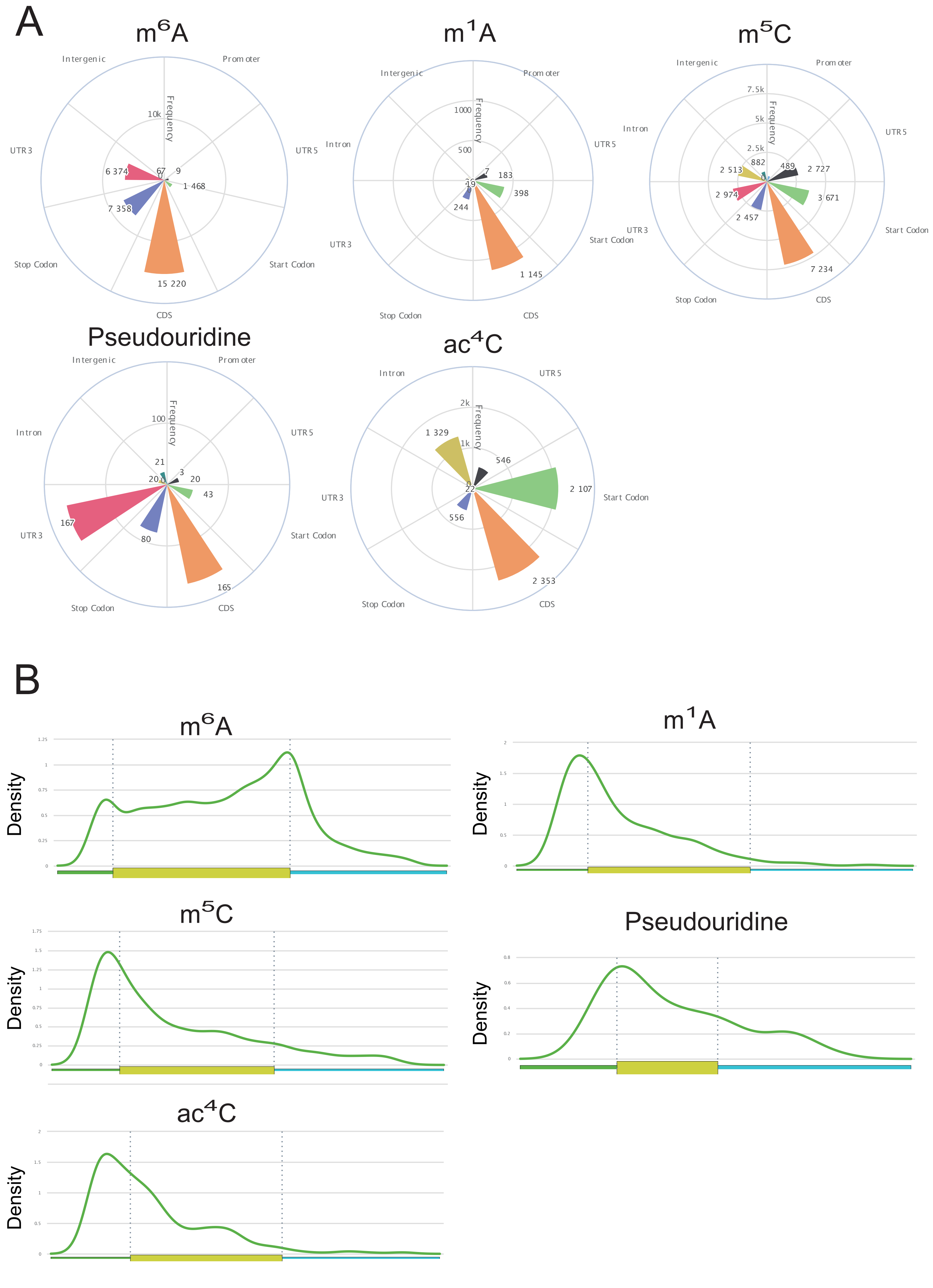
A
m^{6}A
Intergenic
Promoter
UTR3
UTR5
Frequency
10k
6 374
67
9
1 468
7 358
15 220
Stop Codon
Start Codon
CDS
m^{1}A
Intergenic
Promoter
Intron
UTR5
1000
500
7
183
398
244
1 145
UTR3
Start Codon
Stop Codon
CDS
m^{5}C
Intergenic
Promoter
Intron
UTR5
7.5k
5k
2.5k
882
489
2 727
2 513
2 974
3 671
2 457
7 234
UTR3
Start Codon
Stop Codon
CDS
Pseudouridine
Intergenic
Promoter
Intron
UTR5
100
21
3
20
43
167
80
165
UTR3
Start Codon
Stop Codon
CDS
ac^{4}C
Intron
UTR5
2k
1k
1 329
546
2 107
556
22
2 353
UTR3
Start Codon
Stop Codon
CDS
B
m^{6}A
Density
m^{1}A
Density
m^{5}C
Density
Pseudouridine
Density
ac^{4}C
Density

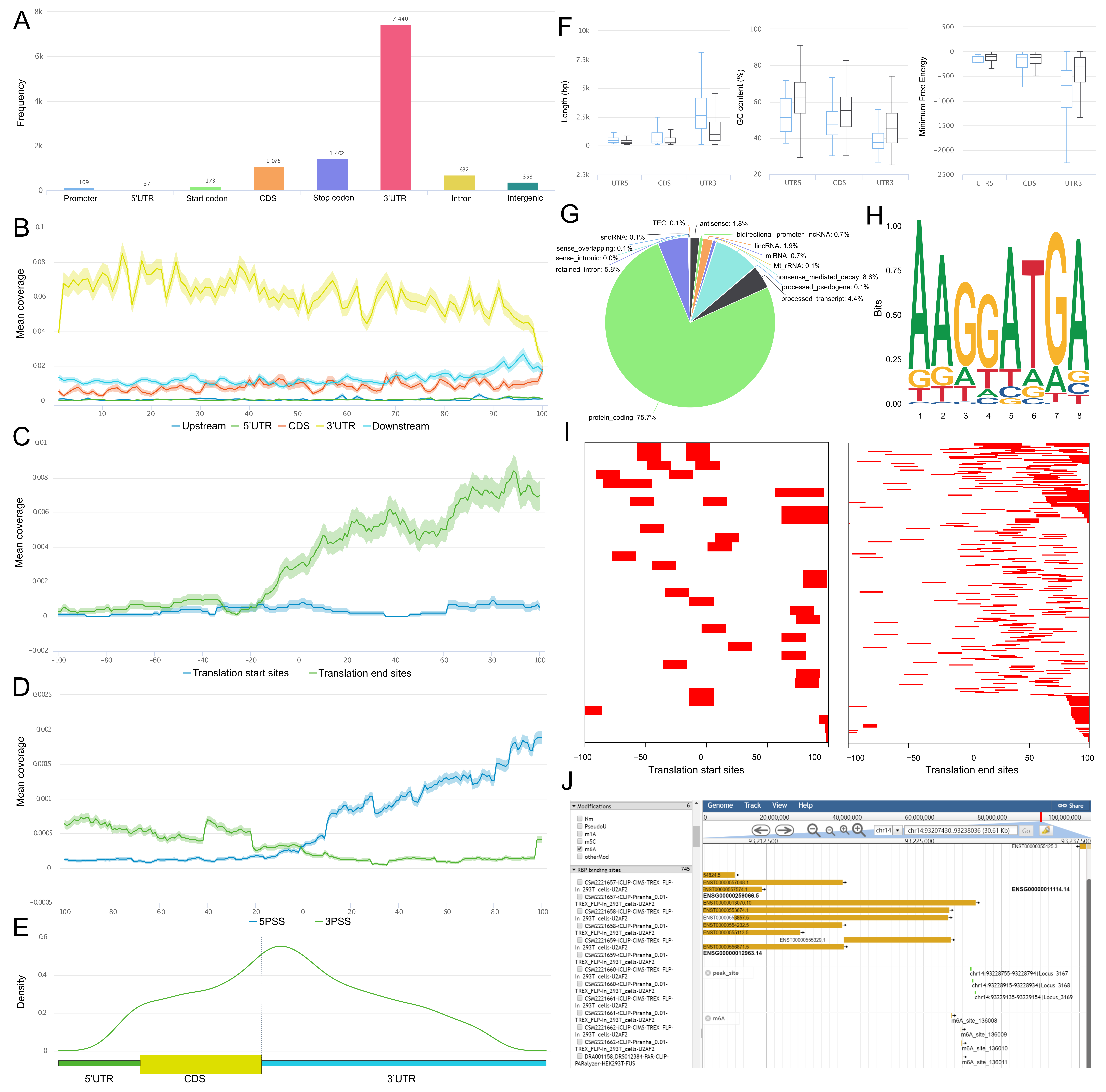

A
Frequency
8k
6k
4k
2k
0
109
37
173
1 075
1 402
7 440
682
353
Promoter
5'UTR
Start codon
CDS
Stop codon
3'UTR
Intron
Intergenic
B
Mean coverage
Upstream
5'UTR
CDS
3'UTR
Downstream
C
Mean coverage
Translation start sites
Translation end sites
D
Mean coverage
5PSS
3PSS
E
Density
5'UTR
CDS
3'UTR
F
Length (bp)
GC content (%)
Minimum Free Energy
UTR5
CDS
UTR3
G
TEC: 0.1%
antisense: 1.8%
bidirectional_promoter_lncRNA: 0.7%
snoRNA: 0.1%
lincRNA: 1.9%
sense_overlapping: 0.1%
miRNA: 0.7%
sense_intronic: 0.0%
Mt_rRNA: 0.1%
retained_intron: 5.8%
nonsense_mediated_decay: 8.6%
processed_psedogene: 0.1%
processed_transcript: 4.4%
protein_coding: 75.7%
H
Bits
1.00
0.75
0.50
0.25
0.00
I
Translation start sites
Translation end sites
J
Modifications
Nm
PseudoU
m1A
m5C
m6A
otherMod
RBP binding sites
Genome
Track
View
Help
Share
chr14
chr14:93207430..93238036 (30.61 Kb)
Go
ENSG00000011114.14
ENSG00000259066.5
ENSG00000012963.14
peak_site
m6A
chr14:93228755-93228794|Locus_3167
chr14:93228915-93228934|Locus_3168
chr14:93229135-93229154|Locus_3169
m6A_site_136008
m6A_site_136009
m6A_site_136010
m6A_site_136011
