# Supplementary material for: RNAmod: an integrated system for the annotation of mRNA modifications
Source: Nucleic Acids Res. 2019 May 31;47(W1):W548–55. doi: 10.1093/nar/gkz479 (PMC6602476; doi:10.1093/nar/gkz479)
Supplement: gkz479_Supplemental_Files [file gkz479_supplemental_files.zip › Table S1.docx]

**Table S1. The available tools for RNA modification prediction and annotation**

| **Function** | | **Name** | **Links** | **Type** | **Ref** |
| --- | --- | --- | --- | --- | --- |
| **Modification prediction tools** | | | | | |
| m^6^A | WHISTLE | | <http://180.208.58.19/whistle/index.html> | web | ([1](#_ENREF_1)) |
| m^6^A | DeepM6ASeq | | <https://github.com/rreybeyb/DeepM6ASeq> | local | ([2](#_ENREF_2)) |
| m^6^A | BERMP | | <http://www.bioinfogo.org/bermp/> | web | ([3](#_ENREF_3)) |
| m^6^A | SRAMP | | <http://www.cuilab.cn/sramp> | web | ([4](#_ENREF_4)) |
| m^6^A | RNAMethPre | | <http://bioinfo.tsinghua.edu.cn/RNAMethPre/index.html> | web | ([5](#_ENREF_5)) |
| m^6^A | RFAthM6A | | <https://github.com/nongdaxiaofeng/RFAthM6A> | local | ([6](#_ENREF_6)) |
| m6A | iRNA(m6A)-PseDNC | | <http://lin-group.cn/server/iRNA(m6A)-PseDNC.php> | web | ([7](#_ENREF_7)) |
| m^6^A | iRNA-Methyl | | <http://lin-group.cn/server/iRNA-Methyl> | web | ([8](#_ENREF_8)) |
| m^6^A | MethyRNA | | <http://lin-group.cn/server/MethyRNA> | web | ([9](#_ENREF_9)) |
| m6A | RAM-NPPS | | <http://server.malab.cn/RAM-NPPS/index.jsp> | web | ([10](#_ENREF_10)) |
| m^6^A | HMpre | | <https://github.com/Zhixun-Zhao/HMpre> | local | ([11](#_ENREF_11)) |
| m^6^A | AthMethPre | | <http://bioinfo.tsinghua.edu.cn/AthMethPre/index.html> | web | ([12](#_ENREF_12)) |
| m^6^A | M6APred-EL | | <http://server.malab.cn/M6APred-EL> | web | ([13](#_ENREF_13)) |
| m^6^A | pRNAm-PC | | <http://www.jci-bioinfo.cn/pRNAm-PC> | web | ([14](#_ENREF_14)) |
| m5C | PEA-m5C | | <https://github.com/cma2015/PEA-m5C> | local | ([15](#_ENREF_15)) |
| m5C | M5C-HPCR | | <http://cslab.just.edu.cn:8080/M5C-HPCR> | web | ([16](#_ENREF_16)) |
| m5C | RNAm5Cfinder | | <http://www.rnanut.net/rnam5cfinder> | web | ([17](#_ENREF_17)) |
| 2'-O-methylation | iRNA-2methyl | | <http://www.jci-bioinfo.cn/iRNA-2methyl> | web | ([18](#_ENREF_18)) |
| pseudouridine | PseUI | | <http://zhulab.ahu.edu.cn/PseUI/> | web | ([19](#_ENREF_19)) |
| pseudouridine | PPUS | | <http://lyh.pkmu.cn/ppus/> | web | ([20](#_ENREF_20)) |
| Diverse modifications | HAMR | | <https://www.lisanwanglab.org/hamr> | web | ([21](#_ENREF_21)) |
| **Modification annotation tools** | | | | | |
|  | |  |  |  |  |
| Metagene plot | | Guitar | <https://bioconductor.org/packages/release/bioc/html/Guitar.html> | R package | ([22](#_ENREF_22)) |
| Metagene plot | | MetaPlotR | <https://github.com/olarerin/metaPlotR> | local | ([23](#_ENREF_23)) |
| Metagene plot and RNA features annotation | | RNAModR | <https://github.com/mevers/RNAModR> | local | ([24](#_ENREF_24)) |
| Metagene plot and RNA features annotation | | RCAS | <https://bioconductor.org/packages/release/bioc/html/RCAS.html> | R package | ([25](#_ENREF_25)) |
| Metagene plot and basic gene annotation | | modTools | <http://rna.sysu.edu.cn/rmbase> | web | ([26](#_ENREF_26)) |

1. Zhang, Q., Chen, K., Wu, X., Wei, Z., Rong, R., Lu, Z., Meng, J., de Magalhães, J.P., Su, J. and Rigden, D.J. (2019) WHISTLE: a high-accuracy map of the human N6-methyladenosine (m6A) epitranscriptome predicted using a machine learning approach.

2. Zhang, Y. and Hamada, M. (2018) DeepM6ASeq: prediction and characterization of m6A-containing sequences using deep learning. *BMC bioinformatics*, **19**, 524.

3. Huang, Y., He, N., Chen, Y., Chen, Z. and Li, L. (2018) BERMP: a cross-species classifier for predicting m(6)A sites by integrating a deep learning algorithm and a random forest approach. *International journal of biological sciences*, **14**, 1669-1677.

4. Zhou, Y., Zeng, P., Li, Y.H., Zhang, Z. and Cui, Q. (2016) SRAMP: prediction of mammalian N6-methyladenosine (m6A) sites based on sequence-derived features. *Nucleic acids research*, **44**, e91.

5. Xiang, S., Liu, K., Yan, Z., Zhang, Y. and Sun, Z. (2016) RNAMethPre: A Web Server for the Prediction and Query of mRNA m6A Sites. *PloS one*, **11**, e0162707.

6. Wang, X. and Yan, R. (2018) RFAthM6A: a new tool for predicting m(6)A sites in Arabidopsis thaliana. *Plant molecular biology*, **96**, 327-337.

7. Chen, W., Ding, H., Zhou, X., Lin, H. and Chou, K.C. (2018) iRNA(m6A)-PseDNC: Identifying N(6)-methyladenosine sites using pseudo dinucleotide composition. *Analytical biochemistry*, **561-562**, 59-65.

8. Chen, W., Feng, P., Ding, H., Lin, H. and Chou, K.C. (2015) iRNA-Methyl: Identifying N(6)-methyladenosine sites using pseudo nucleotide composition. *Analytical biochemistry*, **490**, 26-33.

9. Chen, W., Tang, H. and Lin, H. (2017) MethyRNA: a web server for identification of N(6)-methyladenosine sites. *Journal of biomolecular structure & dynamics*, **35**, 683-687.

10. Xing, P., Su, R., Guo, F. and Wei, L. (2017) Identifying N(6)-methyladenosine sites using multi-interval nucleotide pair position specificity and support vector machine. *Scientific reports*, **7**, 46757.

11. Zhao, Z., Peng, H., Lan, C., Zheng, Y., Fang, L. and Li, J. (2018) Imbalance learning for the prediction of N(6)-Methylation sites in mRNAs. *BMC genomics*, **19**, 574.

12. Xiang, S., Yan, Z., Liu, K., Zhang, Y. and Sun, Z. (2016) AthMethPre: a web server for the prediction and query of mRNA m(6)A sites in Arabidopsis thaliana. *Molecular bioSystems*, **12**, 3333-3337.

13. Wei, L., Chen, H. and Su, R. (2018) M6APred-EL: A Sequence-Based Predictor for Identifying N6-methyladenosine Sites Using Ensemble Learning. *Molecular therapy. Nucleic acids*, **12**, 635-644.

14. Liu, Z., Xiao, X., Yu, D.J., Jia, J., Qiu, W.R. and Chou, K.C. (2016) pRNAm-PC: Predicting N(6)-methyladenosine sites in RNA sequences via physical-chemical properties. *Analytical biochemistry*, **497**, 60-67.

15. Song, J., Zhai, J., Bian, E., Song, Y., Yu, J. and Ma, C. (2018) Transcriptome-Wide Annotation of m(5)C RNA Modifications Using Machine Learning. *Frontiers in plant science*, **9**, 519.

16. Zhang, M., Xu, Y., Li, L., Liu, Z., Yang, X. and Yu, D.J. (2018) Accurate RNA 5-methylcytosine site prediction based on heuristic physical-chemical properties reduction and classifier ensemble. *Analytical biochemistry*, **550**, 41-48.

17. Li, J., Huang, Y., Yang, X. and Zhou, Y. (2018) RNAm5Cfinder: A Web-server for Predicting RNA 5-methylcytosine (m5C) Sites Based on Random Forest. *Scientific reports*, **8**, 17299.

18. Qiu, W.R., Jiang, S.Y., Sun, B.Q., Xiao, X., Cheng, X. and Chou, K.C. (2017) iRNA-2methyl: Identify RNA 2'-O-methylation Sites by Incorporating Sequence-Coupled Effects into General PseKNC and Ensemble Classifier. *Med Chem*, **13**, 734-743.

19. He, J., Fang, T., Zhang, Z., Huang, B., Zhu, X. and Xiong, Y. (2018) PseUI: Pseudouridine sites identification based on RNA sequence information. *BMC bioinformatics*, **19**, 306.

20. Li, Y.H., Zhang, G. and Cui, Q. (2015) PPUS: a web server to predict PUS-specific pseudouridine sites. *Bioinformatics*, **31**, 3362-3364.

21. Ryvkin, P., Leung, Y.Y., Silverman, I.M., Childress, M., Valladares, O., Dragomir, I., Gregory, B.D. and Wang, L.S. (2013) HAMR: high-throughput annotation of modified ribonucleotides. *RNA*, **19**, 1684-1692.

22. Cui, X., Wei, Z., Zhang, L., Liu, H., Sun, L., Zhang, S.W., Huang, Y. and Meng, J. (2016) Guitar: An R/Bioconductor Package for Gene Annotation Guided Transcriptomic Analysis of RNA-Related Genomic Features. *BioMed research international*, **2016**, 8367534.

23. Olarerin-George, A.O. and Jaffrey, S.R. (2017) MetaPlotR: a Perl/R pipeline for plotting metagenes of nucleotide modifications and other transcriptomic sites. *Bioinformatics*, **33**, 1563-1564.

24. Evers, M., Shafik, A., Schumann, U. and Preiss, T. (2016) RNAModR: Functional analysis of mRNA modifications in R. *bioRxiv*, 080051.

25. Uyar, B., Yusuf, D., Wurmus, R., Rajewsky, N., Ohler, U. and Akalin, A. (2017) RCAS: an RNA centric annotation system for transcriptome-wide regions of interest. *Nucleic acids research*, **45**, e91.

26. Xuan, J.J., Sun, W.J., Lin, P.H., Zhou, K.R., Liu, S., Zheng, L.L., Qu, L.H. and Yang, J.H. (2018) RMBase v2.0: deciphering the map of RNA modifications from epitranscriptome sequencing data. *Nucleic acids research*, **46**, D327-D334.
